# Evaluation of preoperative duloxetine use for postoperative analgesia following laparoscopic cholecystectomy: A randomized controlled trial

**DOI:** 10.3389/fphar.2022.944392

**Published:** 2022-09-29

**Authors:** Noha O. Mansour, Sherif Boraii, Mohamed Hassan Elnaem, Mahmoud E. Elrggal, Tamer Omar, Amr Abdelraouf, Doaa H. Abdelaziz

**Affiliations:** ^1^ Clinical Pharmacy and Pharmacy Practice Department, Faculty of Pharmacy, Mansoura University, Mansoura, Egypt; ^2^ Department of Hepatobiliary Pancreatic Surgery, The National Hepatology and Tropical Medicine Research Institute, Cairo, Egypt; ^3^ Quality Use of Medicines Research Group, Department of Pharmacy Practice, Faculty of Pharmacy, International Islamic University Malaysia, Kuantan, Pahang, Malaysia; ^4^ Department of Clinical Pharmacy, College of Pharmacy, Umm Al-Qura University, Mecca, Saudi Arabia; ^5^ Department of Anesthesia, The National Hepatology and Tropical Medicine Research Institute, Cairo, Egypt; ^6^ Department of Clinical Pharmacy, The National Hepatology and Tropical Medicine Research Institute, Cairo, Egypt; ^7^ Pharmacy Practice and Clinical Pharmacy Department, Faculty of Pharmacy, Future University in Egypt, Cairo, Egypt

**Keywords:** pain, postoperative, laparoscopic cholecystectomy, duloxetine, analgesia

## Abstract

**Background:** The pain pattern after laparoscopic cholecystectomy (LC) is complex and distinct from postoperative pain after other laparoscopic procedures, suggesting that procedure-specific optimal analgesic management plans should be proposed. Duloxetine, a non-opioid neuromodulator, has been widely used to manage pain with dual central and peripheral analgesic properties.

**Aims:** To assess the effect of preoperative administration of duloxetine compared to placebo on postoperative pain control in patients undergoing LC.

**Patients and Methods:** This study was a randomized, parallel-group, placebo-controlled, double-blinded study performed on patients undergoing LC. Patients were randomly divided into two groups of 30 each on the day of surgery in the preoperative holding area, using a computer-generated random number to receive 60 mg duloxetine as a single oral dose 2 h before the procedure or placebo. The primary outcome was the difference in the mean of visual analogue scale (VAS) scores between the two studied groups, as measured by the area under the curve (AUC) of the VAS scores.

**Results:** The derived AUC of VAS scores in the duloxetine group (757.89 ± 326.01 mm × h) was significantly lower than that calculated for the control group (1005.1 ± 432.5 mm × h). The mean postoperative VAS scores recorded at 4 and 24 h were statistically different between the study groups (*p* = 0.041 and 0.003, respectively). As observed in the survival curve analysis, there was no significant difference (*p* = 0.665) for the time until the patient’s first request for rescue medications in the two groups. The frequency of postoperative nausea and vomiting (PONV) was lower in patients of the duloxetine group than that recorded in those allocated to the control group at 8 and 24-h time intervals (*p* = 0.734 and 0.572, respectively).

**Conclusion:** Preoperative use of duloxetine reduces postoperative pain significantly compared with placebo. In addition, its use is associated with a reduction in PONV. These preliminary findings suggest that duloxetine could play a role in the acute preoperative period for patients undergoing LC.

**Clinical Trial Registration:** [https://clinicaltrials.gov/ct2/show/NCT05115123, identifier NCT05115123],

## 1 Introduction

Laparoscopic cholecystectomy (LC) has become the benchmark for the treatment of symptomatic gallbladder diseases (Medina-Diaz-Cortés et al., 2022). Existing preferences for laparoscopic cholecystectomy are justified by the quicker recovery of LC patients compared to those undergoing open cholecystectomy (Keus et al., 2010). Despite LC having minimal invasiveness, the post-procedural acute pain might delay recovery and require an overnight hospital stay on the day of surgery in 17–41% of the patients (Bisgaard T. et al., 2001; Lau and Brooks 2001; Bisgaard Thue and Warltier 2006; Gurusamy et al., 2008; Vaughan et al., 2013; Ko-Iam et al., 2017). Controlling postoperative pain still poses a substantial challenge for clinicians, particularly in subsets of patient populations with a high risk for increased severity of post-surgical pain (Bisgaard. et al., 2001; Lee et al., 2022).

Pain pattern after LC is complex and distinct from postoperative pain after other laparoscopic procedures (Bisgaard. 2006), suggesting that procedure-specific optimal analgesic management plans should be proposed (Barazanchi et al., 2018). The preoperative inflammation may sensitize the central nervous system and worsen the pain (Wills and Hunt 2000). Different mechanisms mediate postoperative pain at multiple neural sites, suggesting the need for combined analgesic (Pogatzki-Zahn et al., 2017). Multimodal analgesia techniques, defined as the use of more than one pharmacological class of analgesic medication targeting different receptors, have been highly advocated to relieve post-LC pain. This approach facilitates synergism between different medications, minimizes associated adverse effects, and enhances clinical outcomes (Barazanchi et al., 2018; Schwenk and Mariano 2018).

There are numerous arguments in favor of optimally tailored LC analgesic protocols (Bisgaard Thue and Warltier 2006). However, the most substantial evidence emerges from a systematic review of data using 258 trials done by the procedure-specific postoperative pain management (PROSPECT) working group (Barazanchi et al., 2018). The PROSPECT updated report recommended optimal post-LC pain control by a basic analgesic regimen comprised of combined acetaminophen/nonsteroidal inflammatory drugs (NSAIDs). This regimen should be started preoperatively and continued in the postoperative phase to prevent peripheral pain (Barazanchi et al., 2018). In patients with severe pain, centrally acting opioids are the standard option for providing rescue analgesia post LC (Michaloliakou et al., 1996; Schwenk and Mariano 2018). However, in response to the growing domestic opioid crisis, drug shortage, and greater awareness of opioid-related adverse events, attention has shifted to non-opioids as the basis for pain management (Schwenk and Mariano 2018).

Consequently, there has been a growing interest in novel opioid-free preventive methods specifically for pain prevention in patients undergoing LC. In this regard, gabapentinoids were among the most clinically investigated options, but there was a lack of consensus about their benefit in patients undergoing LC. Positive reported outcomes were detected in some trials (Bekawi et al., 2014; Esmat and Farag 2015) while not in others (Chang et al., 2009; Gurunathan et al., 2016). So far, despite this inconsistent evidence and unfavorable increased postoperative sedation level (Mishriky et al., 2015), the use of gabapentin as an alternative modality has been recommended by the PROSPECT report (Barazanchi et al., 2018). Thus, the need to find more efficacious and tolerable options is crucially demanded, especially when “basic” analgesic techniques are not feasible, as in patients with contraindications to NSAIDs or when an inadequate response is predicted due to the presence of patient-related risk factors for severe pain.

Duloxetine, a non-opioid neuromodulator, has been widely used to manage neuropathic pain (Quilici et al., 2009). It possesses dual central and peripheral analgesic properties (Onuţu 2015). These effects are either due to increased neurotransmission of serotonin and norepinephrine in the descending inhibitory pain pathways of the brain and spinal cord (Onuţu 2015; Zorrilla-Vaca et al., 2019) or through the downstream inhibitory effect of sodium channels (Wang et al., 2010). In terms of safety, duloxetine showed excellent profile as there was no significant difference in the incidence of side effects between duloxetine and placebo in acute pain studies. Moreover, duloxetine decreased incidence of postoperative nausea/vomiting (PONV), a commonly occurring event following different surgeries (Zorrilla-Vaca et al., 2019).

Compiled evidence arises from a recent meta-analysis that linked perioperative duloxetine use to a significant reduction in acute pain at different time intervals ranging from 4 to 48 h post-procedure (Zorrilla-Vaca et al., 2019). However, the results of this analysis should be interpreted with extreme caution due to multiple serious limitations. First, the observed heterogeneity in the investigated doses and durations of duloxetine use might have impacted the results. Second, the inclusion of trials with patient populations undergoing different types of procedures that ranged from major surgeries to minor invasive laparoscopic ones. Despite favorable analgesic outcomes reported in the meta-analysis, this could not be generalized to pain outcomes in LC as none of their included studies enrolled this particular patient population. The use of analgesic regimens that include at least one opioid drug in most studies is another notable limitation that impedes its generalizability. In light of the increasing global efforts to spare opioids, the use of duloxetine as a part of an opioid-free multimodal analgesic regimen in LC procedures needs investigation. Therefore, the aim of the present study was to assess the effect of preoperative administration of duloxetine compared to placebo on postoperative pain control in patients undergoing LC.

## 2 Patients and methods

### 2.1 Study design

This study was a randomized, parallel-group, placebo-controlled, double-blinded study performed on patients undergoing LC. Institutional review board approval was obtained from the National Hepatology and Tropical Medicine Research Institute (NHTMRI), where the study was conducted. The trial protocol was registered prior to patient enrolment at clinicaltrials.gov (NCT05115123). The study was performed according to the Declaration of Helsinki and was reported according to the Consolidated Standards of Reporting Trials (CONSORT) statement. Each included patient signed a written informed consent before enrolment.

### 2.2 Subjects

All patients between the ages of 18 and 70 who were admitted to NHTMRI for elective LC were evaluated for eligibility during the pre-assessment clinic visit. Exclusion criteria included liver or renal dysfunction, chronic pain other than cholelithiasis, daily corticosteroid use, or a history of duloxetine allergy. Patients with cognitive, psychological, or communication disorders, those who had received analgesics or sedatives in the 24 h prior to surgery, and pregnant or nursing women were all excluded.

### 2.3 Interventions

Patients were randomly divided into two groups of 30 each on the day of surgery in the preoperative holding area, using a computer-generated random number table as follows:

• The duloxetine group administered 60 mg of duloxetine capsule as a single oral dose 2 h before the procedure.
• Patients in the control group were given a capsule identical in size and color to the experimental ones 2 h before surgery. In a set of opaque envelopes, the details of group assignments were kept. The pharmacist who dispensed the drugs after opening the envelope was not a part of the outcome evaluation process. Both the patient and the researcher who observed the patient’s outcome were unaware of the patient’s group assignment.

Intraoperative procedures.

Anesthesia was induced with fentanyl 2 μg/kg, propofol 2 mg/kg and atracurium 0.5 mg/kg. The lungs were mechanically ventilated using the circle system with a 50% mixture of oxygen with air to maintain end- tidal carbon dioxide between 35 and 45 mmHg. At the end of the surgery, the remaining carbon dioxide in the peritoneal cavity was expelled by abdominal decompression. The residual neuromuscular block was antagonized with atropine 0.01 mg/kg and neostigmine 0.04 mg/kg. Neither supplemental analgesics/pre-medications nor local anesthesia were administered either during or before the end of the operation. Duration of operation was documented for each patient. The patients were then moved to the post-anesthesia care unit where the standard pain protocol was initiated. The standard analgesia composed of 1-gram IV acetaminophen every 8 h. A-75 mg intramuscular injection of diclofenac sodium was given to patients who recorded a visual analogue scale (VAS) score of ≥ 70 mm and requested rescue analgesia. No prophylactic antiemetics were given to the patients before the end of the procedures.

### 2.4 Primary outcome

#### 2.4.1 Pain assessment: Visual analogue scale

Each patient received instruction on calculating their postoperative pain using the 100 mm VAS scale before surgery (0 indicated no pain, 100 denoted the most severe pain) (Aun et al., 1986). The patients were instructed to place a mark on the line corresponding to their perceived current condition. The distance (mm) between the beginning of the horizontal line and this mark represented the degree of pain perception. Pain scores were recorded by outcome evaluators who were blinded to study interventions at the inpatient unit at 2, 4, 8, 12, and 24-h following surgery. The primary outcome was the difference in the mean of VAS scores between the two studied groups, as measured by the area under the curve (AUC) of the VAS scores. Individual VAS scores were (virtually) plotted as a curve in which the *x*-axis represented evaluation time from baseline (2 h) to 24 h post-LC, and the *y*-axis represented the VAS score. Using this approach, AUC for each assessment point (trapezoids) was calculated and added together, resulting in an overall VAS AUC score (mm × hr.) that was compared across the treatment groups (Damman et al., 2020). Time to first rescue analgesic request, cumulative consumption of rescue analgesics 24 h postoperatively in mg, and time to unassisted mobilization were also recorded outcomes.

#### 2.4.2 Secondary outcomes

##### 2.4.2.1 Postoperative nausea and vomiting grading and numeric sedation scale

Using a 4-point scale, the frequency of PONV was recorded in the surgery ward after 8 and 24 h for each patient (Table 1). Ondansetron 4 mg IV injection was used to treat moderate to severe nausea and vomiting. The patient’s conscious level was observed and graded using the NSS, which was reported at 2, 4, 8, 12, and 24 h postoperatively on a 5-point numerical scale. (Table 1) (Bekawi et al., 2014).

**TABLE 1:** Sedation and nausea/vomiting scales.

PONV Scores	
(0) None	No nausea, vomiting, or retching
(1) Mild	Happened once
(2) Moderate	Happened 2–3 times
(3) Severe	Continuous or ≥3 times
**Numeric Sedation Scale (NSS)**	
1	Completely awake
2	Awake but drowsy
3	Asleep but responsive to verbal order
4	Asleep but responsive to tactile stimulus
5	Asleep and nonresponsive to any stimuli

PONV, postoperative nausea and vomiting grading.

#### 2.4.3 Safety assessment

Common side effects related to duloxetine were recorded in the post anaesthesia care unit and the ward.

### 2.5 Sample size

No previous studies were available to estimate the actual effect size of preoperative duloxetine use on VAS scores in LC patients. This study was designed as a pilot one; based on the previous studies in acute pain management, a large effect size in the primary outcome measure was assumed (Altiparmak et al., 2018; Zorrilla-Vaca et al., 2019). The required sample size was calculated using G*Power software version 3.1.0 (Institut fur Experimentelle Psychologie, Heinrich Heine Universitat, Dusseldorf, Germany). Assuming *α* error = 0.05 (2-tailed) and a power of 0.80, 26 participants were needed per treatment group. Considering a 15% dropout rate, sample size of 30 subjects in each group was randomized.

Statistical analysis was done using IBM SPSS^®^ Statistics version 26 (IBM^®^ Corp., Armonk, NY, United States). The mean and standard deviation were used to express numerical data. Qualitative data were expressed as frequency and percentage. The Skewness-Kurtosis and Shapiro-Wilk tests were used to check for normality, but the data were not normally distributed, so nonparametric tests were used. A comparison between two groups concerning continuous variables was made using the Mann-Whitney test. When applicable, categorical variables were analysed using Pearson chi-square tests or Fisher’s exact tests. A comparison of each group over time was carried out using the Freidman test. Using a log-rank test, time to event analysis was compared with the Kaplan-Meier survival curve. In time to first rescue analgesia and time to unassisted mobilization, survival time was defined as the time (hours) from the end of the operation to the event. In all cases, statistical significance was defined as *p* < 0.05.

## 3 Results

### 3.1 Patients and baseline analysis

**FIGURE 1:**
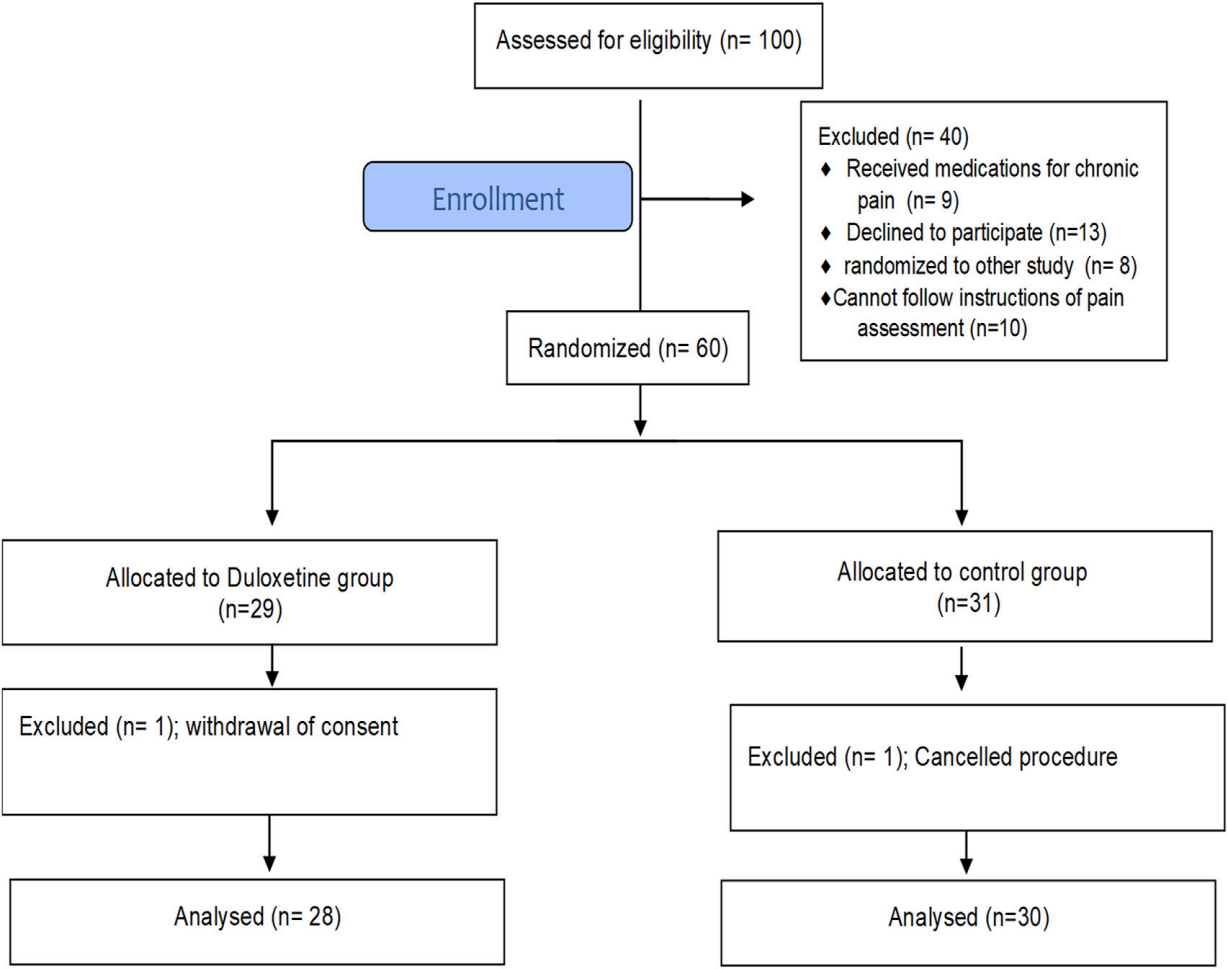
CONSORT flow diagram showing the flow of patients throughout the study.

A total of 100 patients scheduled for elective LC were screened for eligibility from March to April 2022. Sixty patients, of both sexes, with American Society of Anesthesiology (ASA) grade I to II, met the inclusion criteria and were randomly assigned to one of the study groups. Figure 1 shows that 58 patients completed the study and were included in the final analysis. At baseline, there was no significant difference between groups in terms of basic demographic data, comorbid conditions, or surgery duration (Table 2).

**TABLE 2:** Baseline demographic and clinical variables of the two study groups.

Parameters	Duloxetine Group (*n* = 29)	Control Group (*n* = 31)	*p*-Value
Age (years)‡	40.79 ± 11.63	39.65 ± 12.79	0.773*
Age Categories n (%)			0.997**¥**
*<40 years*	15 (51.72)	16 (51.61)	
*40–59 years*	12 (41.38)	13 (41.94)	
*≥ 60 years*	2 (6.90)	2 (6.45)	
Weight (kg) ‡	80.79 ± 16.58	74.26 ± 12.93	
BMI Categories n (%)			0.613**¥**
*Underweight*	0 (0)	1 (3.22)	
*Normal weight*	5 (17.24)	8 (25.81)	
*Overweight*	12 (41.38)	12 (38.71)	
*Obese*	12 (41.38)	10 (32.26)	
Gender; n (%)			0.101**¥**
Male	10 (34.48)	5 (16.13)	
Female	19 (65.52)	26 (83.87)	
Smoking Status n (%)			0.111**¥**
*Smokers*	11 (37.93)	6 (19.35)	
*Non-smokers*	18 (62.07)	25 (80.65)	
History of drug/alcohol abuse n (%)	2 (6.89)	1 (3.23)	0.475**+**
SBP (mmHg) ‡	123.72 ± 9.54	121.19 ± 9.53	0.190 *
DBP (mmHg) ‡	79.52 ± 6.32	75.97 ± 7.73	0.073*
Preoperative respiratory rate (Breath/minute) ‡	20.62 ± 2.25	19.81 ± 1.40	0.259*
Preoperative pulse (Beat/minute)‡	81.48 ± 10.79	80.87 ± 12.95	0.917*
Co-morbidities; n (%)			0.899**¥**
*None*	17 (58.62)	19 (61.30)	
*Hypertension*	7 (24.14)	6 (19.35)	
*Hypertension & Diabetes mellitus*	5 (17.24)	6 (19.35)	
ASA status n (%)			0.244**¥**
*I*	21 (72.40)	18 (58.06)	
*II*	8 (27.60)	13 (41.94)	
Duration of surgery (min) ‡	69.40 ± 23.15	74.10 ± 33.36	0.982*

‡ Mean ± standard deviation; BMI, body mass index; ASA, american society of anesthesiology; n, number of patients; min: minutes; ^**¥**^ *p*-values calculated from chi-square test; * *p*-values calculated from Mann-Whitney test; ^**+**^values calculated from Fisher’s exact test.

### 3.2 Pain assessment

The mean VAS scores recorded at 4 and 24 h postoperative were statistically different between the study groups (*p* = 0.041 and 0.003, respectively) (Table 3). Although the mean VAS score of the duloxetine group was lower than that of the control group at 2, 8, and 12 h postoperatively, the difference was not statistically significant (*p* = 0.063, 0.195, and 0.065, respectively).

**TABLE 3:** Pain parameters post LC in the two study groups.

Parameters	Duloxetine Group *n* = 28	Control Group *n* = 30	*p* value
VAS score (mean ± SD) (mm)			
*VAS at 2 h*	49.32 ± 27.62	62.70 ± 25.96	0.063*
*VAS at 4 h*	47.29 ± 23.87	58.30 ± 25.90	0.041*
*VAS at 8 h*	43.36 ± 19.00	52.27 ± 25.11	0.195*
*VAS at 12 h*	31.86 ± 14.80	41.13 ± 20.72	0.065*
*VAS at 24 h*	23.07 ± 17.00	38.23 ± 20.51	0.003*
	0.001†	<0.001†	
Diclofenac intake			
*Patients needed rescue* n (%)	6 (21.42)	7 (23.33)	0.862
*Average cumulative amount (mg)*	87.50 ± 30.62	150.0 ± 61.24	0.073*

*p-values calculated from Mann-Whitney test; †*p*-values calculated from Friedman test, *n* = Number of patients.

**FIGURE 2:**
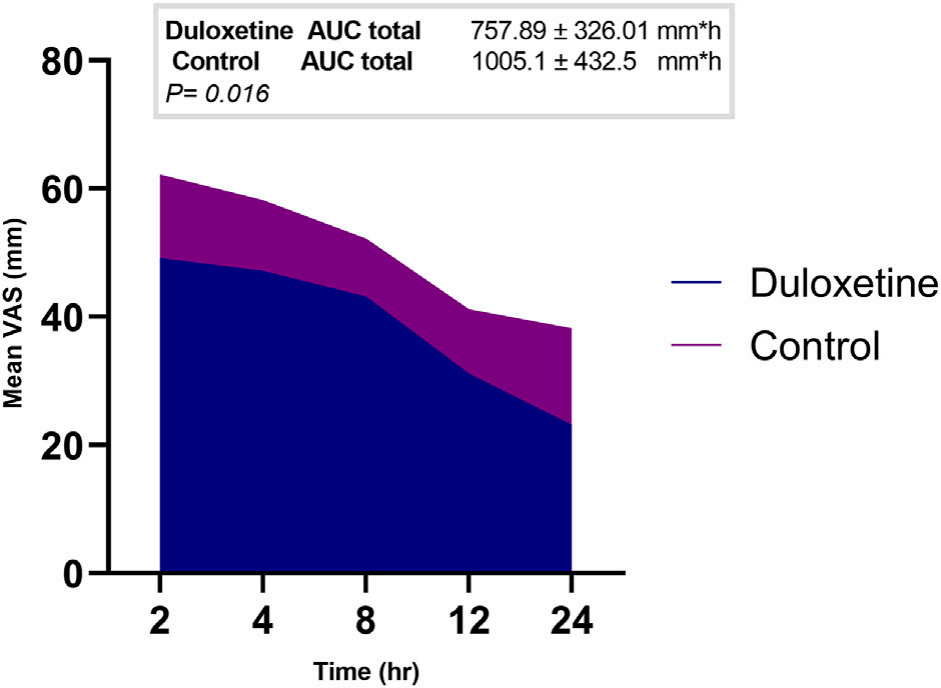
Area graph representing the mean VAS score at the different time intervals in the two groups.

In each study group, a marked decrease in the mean VAS score was observed over time compared to baseline; the duloxetine group had lower mean scores than the control group at all study points (Table 3). In addition, a significantly smaller AUC of VAS score (the primary outcome) was observed in the duloxetine group as compared to that in the control group (757.89 ± 326.01 mm × h vs 1005.1 ± 432.5 mm × h; *p* = 0.016) (Figure 2).

### 3.3 Assessment of recovery and need for rescue analgesics

**FIGURE 3:**
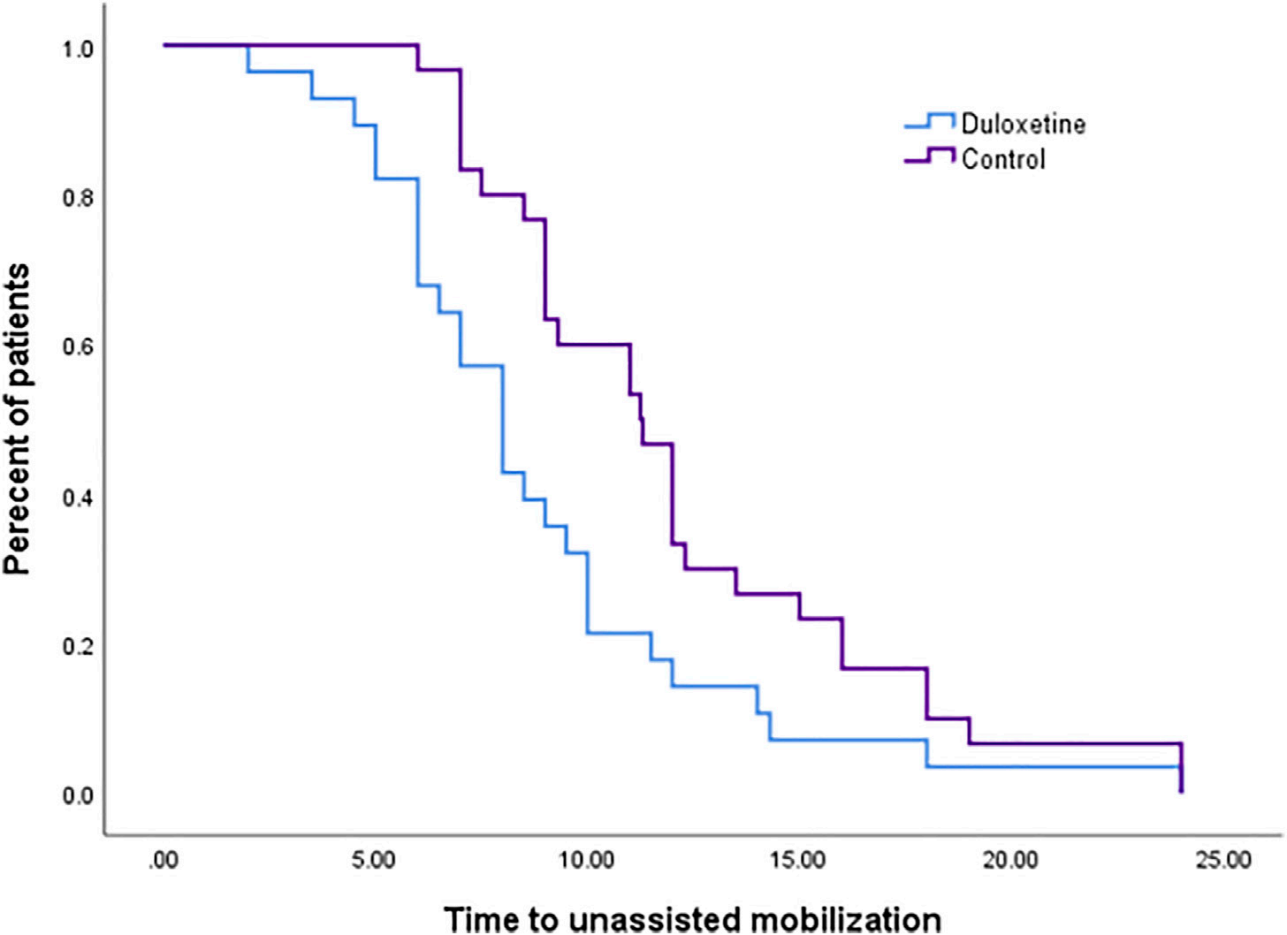
Kaplan-Meier survival curve represents time to unassisted mobilization (*p* = 0.014).

There was no significant difference (*p* = 0.665) for the time until the patient’s first request for rescue medications in the two groups, as observed in the survival curve analysis (Supplementary A). The percentage of patients who requested rescue analgesia in the control group was comparable to that in the duloxetine one (*p* = 0.862); however, higher cumulative doses of analgesia were required in the control group (*p* = 0.073) (Table 3). Enhanced recovery was observed in the duloxetine group from detected significantly shorter time to unassisted mobilization (Figure 3). The median estimated time was 8.00 h (confidence interval (CI):6.72-9.28) versus 9.00 h (CI: 7.88-10.12) in the duloxetine, and control groups, respectively.

### 3.4 PONV assessment

**FIGURE 4:**
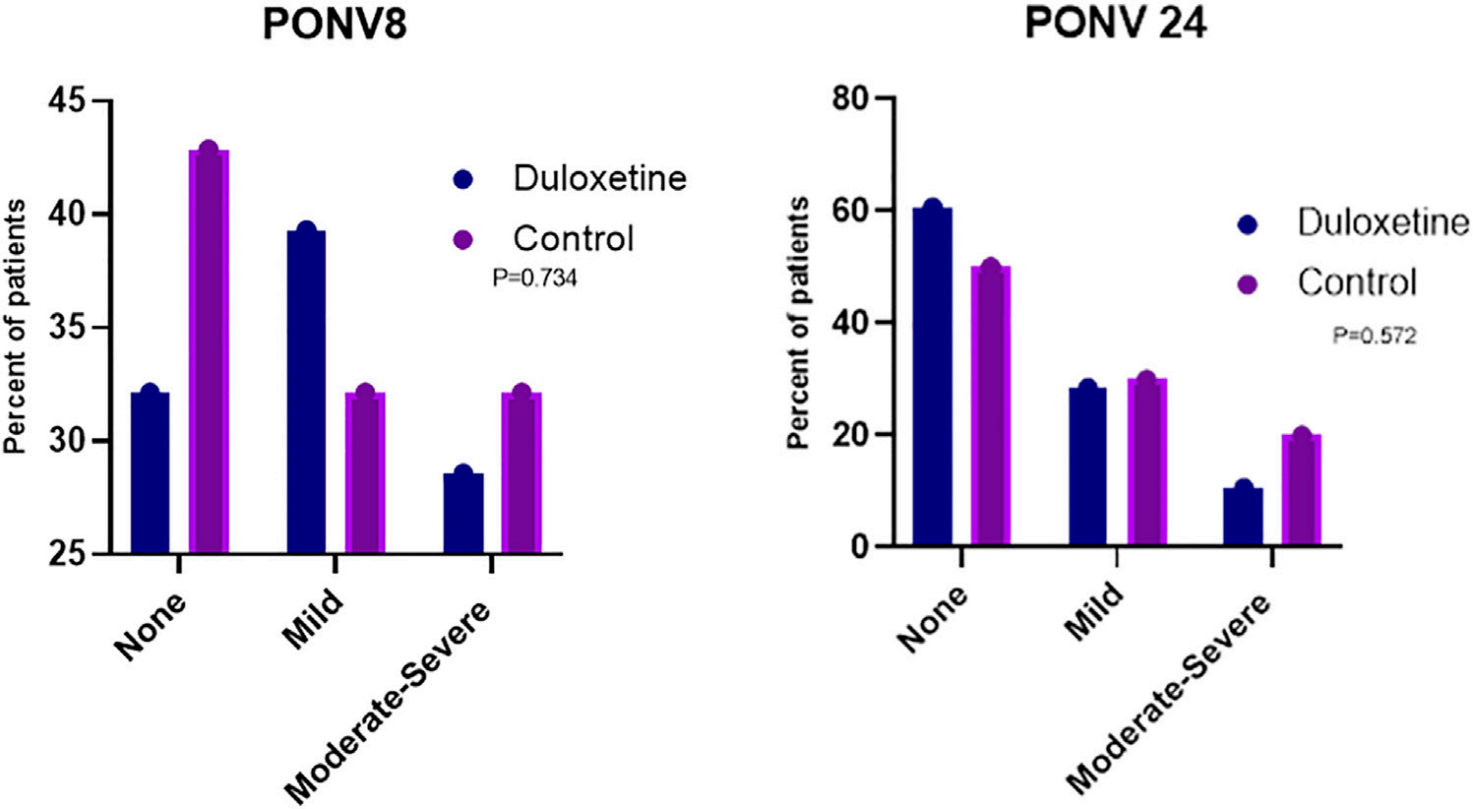
Percentage of patients in the two groups who developed nausea and vomiting with different severities at 8 (PONV 8) and 24 h (PONV 24) post-surgery.

The frequency of PONV was lower in patients of the duloxetine group than that recorded in those allocated to the control group at 8 and 24-h time intervals (*p* = 0.734 and 0.572, respectively). Moderate to severe nausea and vomiting was recorded in 28.57% of patients in the duloxetine group at 8 h, whereas 10% in the same group documented severe PONV at 24 h. In the control group, severe PONV was noted in 32.14% of patients at 8 h and in 20% of patients at 24 h (Figure 4).

### 3.5 Sedation assessment

There was no significant difference between the duloxetine and the control group at the 2, 4, 8, 12, and 24-h intervals regarding NSS score. Sedation scores of both groups consistently improved with time in both groups (Table 4).

**TABLE 4:** The mean sedation scale score in the two groups at the different time intervals.

NSS Score	Duloxetine Group (*n* = 28)	Control Group (*n* = 30)	*p* value
NSS at 2 h n (%)			0.457 ^**¥**^
*Completely awake*	17 (60.71)	21 (70)	
*Awake but drowsy*	11 (39.29)	9 (30)	
NSS at 4 h n (%)			0.369*
*Completely awake*	26 (92.85)	26 (86.67)	
*Awake but drowsy*	2 (7.15)	4 (13.33)	
NSS at 8 h n (%)			0.263*
*Completely awake*	28 (100)	28 (93.33)	
*Awake but drowsy*	0 (0)	2 (6.67)	
NSS at 12 h n (%)			0.749*
*Completely awake*	27 (96.43)	27 (90)	
*Awake but drowsy*	0 (0)	3 (10)	
*Asleep but responsive to verbal order*	1 (3.57)	0 (0)	
NSS at 24 h n (%)			0.517*
*Completely awake*	28 (100)	29 (96.67)	
*Awake but drowsy*	0 (0)	1 (3.33)	

^¥^ *p*-values calculated from chi-square test; *p-values calculated from Fisher’s Exact test. n, Number of Patients, NSS, numeric sedation scale.

### 3.6 Safety outcomes

**FIGURE 5:**
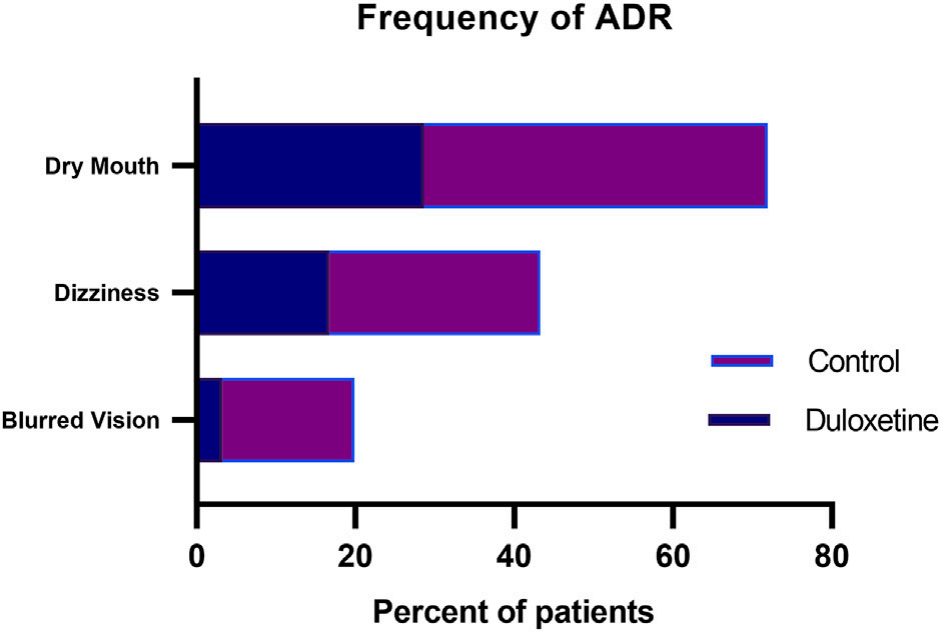
Incidence of the common adverse effects in the two groups.

Among the reported adverse drug reactions (ADR), dry mouth was the most common ADR reported in duloxetine and control groups (28.5%, and 43.3%, respectively) (Figure 5). Dizziness was also frequently reported in both groups. There were no serious adverse reactions, and all patients’ adverse reactions were self-relieved. None of the patients assigned to duloxetine experienced sweating or hypotension, which were only detected in 6.66, and 10% of the control group, respectively. Bradycardia did not occur in any of our included subjects.

## 4 Discussion

The present study results showed that duloxetine administration in the acute preoperative period is associated with a significant reduction in acute postoperative pain compared with placebo without further coincident negative side effects. These findings suggest that duloxetine might play a role in the multimodal pain management options following LC. Multimodal approaches yield not only improved pain scores but also faster discharge and lower cost (Kaye et al., 2019). So far, this study is the first randomized, placebo-controlled trial that has examined the effect of preoperative duloxetine on postoperative pain in patients undergoing LC. This study employed the use of 60 mg duloxetine, the same dose for treating chronic neuropathic pain. The employed strategy is generally based on analyzing effective duloxetine doses in acute pain (Hetta et al., 2020). Daily doses up to 120 mg were studied; however, higher doses were associated with an increased incidence of side effects without additional benefit (Schnabel et al., 2021).

The primary outcome of this study was the difference in pain as measured by comparing the derived AUC of VAS score of the two study groups. The concept of using AUC as a metric for efficacy outcomes has been incorporated into various clinical settings such as asthma, chronic obstructive pulmonary disease (Feldman et al., 2014), and acute heart failure, where the AUC of VAS dyspnea score was evaluated (Teerlink et al., 2013; Damman et al., 2020). In pain clinical trials, AUC reflects the pain pattern over the relevant postoperative period and is preferred rather than selecting a single time point for efficacy assessment. A similar approach was recently followed in evaluating the analgesic effect of a serotonergic receptor blocker by Abdelaziz et al. in patients undergoing LC (Abdelaziz et al., 2021). In the current study, significantly lowered derived AUC of VAS scores in the intervention group was found as compared to that derived from the control group (*p* = 0.016). The primary outcome results reported in the control group of the present study (AUC = 1005.1 mm × h) were deemed comparable with those of the same group in Abdelaziz’s study (AUC = 1114.4 mm × h) (Abdelaziz et al., 2021). This validates our results as both trials used the same approach of basic analgesic regimens and similar intraoperative anesthetic protocol.

Pain intensity attenuation reported in the duloxetine group could be simply explained by the central pain inhibitory action secondary to the potentiation of serotonergic and noradrenergic activities in the central nervous system. Different modes have been postulated for duloxetine to exert its analgesic effects. It acts at the spinal cord level by increasing the level of the neurotransmitters norepinephrine, dopamine, and serotonin in the dorsal horn of the spinal cord. These monoamines activate spinal serotonergic and noradrenergic receptors that potentiate inhibitory descending pain pathways in the spinal cord. Another central mechanism is the activation of the prefrontal cortex, which causes cognitive modulation of pain (Govil et al., 2020). Duloxetine’s peripheral action as a local anaesthetic is due to blockage of neuronal Nav1.7 Na^+^ channel especially in unmyelinated C-type nerve fibers (Zorrilla-Vaca et al., 2019).

Considering this is the first trial to investigate the effect of preemptive analgesia with duloxetine in LC patients, it is reasonable to compare our results with the findings of other surgical procedures. The findings of the present study coincide with the results of Kassim et al. who evaluated the effect of duloxetine on postoperative pain control in patients underwent laparoscopic gynecological surgeries. Duloxetine, in same regimen to that used in the current study, significantly reduced VAS scores, and lessened the requirements for rescue analgesia (Kassim et al., 2018). The benefit of using duloxetine is not only limited to laparoscopic surgeries but also has been extended to other major spinal surgeries. Our findings reproduce the positive evidence reported by Altiparmak et al. who linked lower VAS scores to preoperative duloxetine administration compared to placebo (Altiparmak et al., 2018). Seven randomized controlled trials evaluated the effectiveness of preoperative duloxetine in different procedures and their pooled results stated that duloxetine significantly lowered the pain score at 4 (*p* < 0.001) and 24 h (*p* = 0.005) compared with placebo control. Similar to our results, there was no significant difference in pain scores between both groups at 2 h (Zorrilla-Vaca et al., 2019).

Despite nausea being a stated side effect of duloxetine’s use, particularly in the acute setting (Gartlehner et al., 2009); PONV were notably reduced with duloxetine use as compared to placebo; however, the difference was statistically insignificant. Recent evidence from metanalysis by Schnabel et al. who pooled safety data from clinical trials that employed selective serotonin reuptake inhibitors (SSRIs) use in the management of postoperative pain supports our findings. Schnabel reported that there was no significant difference between patients treated with duloxetine or placebo in terms of incidence of PONV (Schnabel et al., 2021). These findings differed from the conclusions of another meta-analysis (Zorrilla-Vaca et al., 2019), that reported a statistically lower incidence of PONV with duloxetine group compared to placebo. The significantly lower opioid consumption might explain this difference in the duloxetine group throughout their included studies; that perhaps is the key causative factor that underpinned the decrease in PONV incidence.

Enhanced recovery after surgery protocols (ERAS) comprise a combination of various pre-, intra-, and postoperative elements. It integrates different interventions to reduce surgical stress and accelerate recovery in patients undergoing major surgery (Melnyk et al., 2011). These protocols are now well-known to be useful for elective surgeries including LC, as they result in shorter hospital stays without adversely affecting morbidity (Zhang et al., 2020). In the current study, enhanced recovery was seen with duloxetine use as observed from the significantly shorter time to unassisted mobilization in the intervention group compared to the control. The use of preoperative duloxetine could be implemented along with the modified ERAS protocol as a part of opioid free pain management regimen.

Concerning the increased level of sedation, the present study results showed no significant difference between the duloxetine and the control group at different time intervals. Most of the previous studies on perioperative duloxetine use did not evaluate its effect on the level of sedation, except for two studies that yielded conflicting results (Ho et al., 2010; Hetta et al., 2020). Lack of consistency with the finding of (Hetta et al., 2020) might be attributed to the different patient populations, higher examined doses (90 mg duloxetine), and use of morphine as postoperative analgesia.

This study is limited by the small sample size and the method of randomization. It evaluated short-term outcomes of preemptive analgesia with duloxetine; however, long-term outcomes such as rates of developing chronic post-surgical pain are also important and need to be evaluated. In addition, the severity of post-surgical pain might be confounded by some preoperative patient-related factors such as age, anxiety level before surgery, or gender (Hussain et al., 2013; Pereira and Pogatzki-Zahn 2015; Thomazeau et al., 2016; Zorrilla-Vaca et al., 2019; van Dijk et al., 2021). The small sample size and the simple randomization employed in the current study hindered our ability to examine how these confounders might potentially impact the study outcomes. The opioid-sparing effect of duloxetine has not been investigated in this trial because our institutional pain protocol uses diclofenac sodium instead of opioids to rescue severe postoperative pain after LC.

## 5 Conclusion

It has been shown in the current study that preoperative use of duloxetine reduces postoperative pain significantly compared with placebo. In addition, its use is associated with a reduction in PONV. These findings suggest that non-opioid analgesics such as duloxetine could play a role in the acute perioperative period for patients undergoing LC. The data from our study is only preliminary in nature. Further work should explore larger patient samples and different treatment durations. Future multicenter clinical trials with stratified subsets of participants based on possible confounders of severe acute post-surgical pain occurrence are necessary to confirm our findings. Evaluating benefits from preemptive analgesia with duloxetine as a part of multimodal analgesia that integrates novel and promising non-opioid anesthetic modalities administered in the intraoperative and postoperative phases is highly warranted. Investigating the effects of different SSRIs such as venlafaxine and integrating their use to enhance recovery as a part of the ERAS protocol is also recommended. Further trials focusing on safety issues of perioperative SSRIs application are necessary for an appropriate risk-benefit analysis.

## Data Availability

The raw data supporting the conclusions of this article will be made available by the corresponding author upon reasonable request.
